# Exploring physical, subjective and psychological wellbeing profile membership in adolescents: a latent profile analysis

**DOI:** 10.1186/s40359-024-02196-5

**Published:** 2024-12-04

**Authors:** Alexandra Hennessey, Sarah MacQuarrie, Kimberly J. Petersen

**Affiliations:** 1 Manchester Institute of Education, School of Environment, Education and Development, The University of Manchester, Oxford Road, Manchester, M13 9PL UK; 2 School of Education, University of Leeds, Woodhouse, Leeds, LS2 9JT UK

**Keywords:** Wellbeing, Physical activity, Latent profile analysis, Adolescence

## Abstract

**Background:**

Understanding wellbeing in adolescents and within education settings is crucial to supporting young people. However, research defining and exploring wellbeing has typically taken a focus on subjective, psychological, social and emotional domains and has failed to incorporate aspects of physical health and wellbeing. This study aimed to explore how both physical and subjective and psychological wellbeing can be combined to generate different profiles of wellbeing in adolescents, and to understand the characteristics associated with this profile membership.

**Methods:**

366 adolescents aged 11-16yrs (mean age 12.75) from three mainstream secondary schools across England completed an online survey capturing demographic characteristics, physical, subjective and psychological wellbeing, physical activity, emotional literacy, school belonging, and perceptions of learning ability. Latent profile analysis used a data driven approach to explore profiles of wellbeing using physical wellbeing and positive emotional state and positive outlook as predictors of profile membership. To understand profile characteristics demographics, physical activity and educational variables were added as co-variates.

**Results:**

Three profiles were identified, (1) low wellbeing (*n* = 68, 19%) displaying low scores across physical wellbeing, positive emotional state and positive outlook, (2) moderate wellbeing (*n* = 168, 46%) characterised by average levels across physical wellbeing, positive emotional state and positive outlook, and (3) high wellbeing (*n* = 128, 35%) showing high score across physical wellbeing, positive emotional state and positive outlook. Compared to the high wellbeing profile, the moderate and low profiles membership was characterised by being older, being a girl, lower perceived socio-economic status, fewer hours of physical activity a week, and lower emotional literacy, school belonging and perceptions of learning.

**Conclusions:**

The results evidence that physical, subjective and psychological wellbeing are closely inter-related, this finding coupled with increased physical activity in the higher wellbeing group signify physical health and activity are important components of overall wellbeing and should form part of a holistic approach to school wellbeing curriculums.

**Supplementary Information:**

The online version contains supplementary material available at 10.1186/s40359-024-02196-5.

## Background

Understanding wellbeing in the context of education is of ever-increasing need, as is understanding the role of educational institutions in promoting mental health for students. Policy and practice concerning provision for supporting the wellbeing of children and young people in education has taken prime focus as national trends in the UK are reporting a pattern of dips across children and young people’s wellbeing, alongside reduced happiness with their physical health [1]. These trends are echoed internationally, and prevalence rates for depression and anxiety among adolescence is on the rise [2].

The education community is a well-placed to promote both the physical and mental wellbeing of children and young people [1, 3–5]. The paper being reported collected data from adolescents in schools in the UK to better understand the inter-connected facets that relate to their wellbeing. While adolescence is recognised to be a period where some degree of turbulence and change is expected, this can be accompanied by stress and worry [6]. Variation across areas of wellbeing may reflect cumulative difficulties arising post pandemic and data from adolescent themselves is crucial to optimise their environment in education. Such knowledge is vital to understand the role and relationship between physical health, activity and wellbeing so it may feed into the design of education programmes and interventions for future practice. This study therefore first explores profiles of adolescent wellbeing, and second aims to determine how these profiles can be differentiated, to understand possible preventive measures and factors associated with promoting wellbeing in educational settings.

### Models of wellbeing in children and young people

Understanding what constitutes wellbeing is complex, nuanced and multifaceted [7], particularly in adolescent populations at a stage in life with increasing vulnerability to lower levels of wellbeing and developing mental health difficulties [8], and a population at risk of reduced physical activity [9, 10]. A lack of consensus regarding a single definition of wellbeing complicates matters, yet there is a general agreement that wellbeing includes the presence of positive emotions such as contentment and happiness, and the absence of negative emotions such as depression and anxiety [11, 12]. When conceptualising wellbeing two distinctions are typically made, that of hedonic wellbeing or “subjective wellbeing” concerned with the immediate states of happiness, and that of eudaimonic or “psychological wellbeing” concerned with the realisation of potential, personal growth, self-acceptance, life satisfaction [11]. More recent holistic views recognise the overlap and combine subjective and psychological wellbeing and mental health [13] as acknowledged by the World Health Organisation definition: “*state of mental well-being that enables people to cope with the stresses of life*,* realize their abilities*,* learn well and work well*,* and contribute to their community*. *Mental health is an integral component of health and well-being and is more than the absence of mental disorder*” [14]. Recent literature echoes these complexities; identifying the challenges of singling out what is and is not characterised as wellbeing, noting it is an integral component of feeling well adjusted and content across the lifespan [15]. There is a growing body of research exploring how such concepts of wellbeing can be adopted in research with children and young people, and this includes how to robustly measure it [16–18]. A review of ten mental health and wellbeing measures for young people recommended the Stirling Children’s Well-being Scale [16]. The recommendations refer to good implementation and psychometric qualities, positively worded language and theoretical alignment with a multi-faceted conceptualisation of wellbeing across, psychological, emotion and social domains [19]. However, the role of physical health and wellbeing is underplayed and potentially overlooked. Therefore, exploring how physical health, activity and wellbeing relate to subjective and psychological domains of wellbeing is required to better understand, define and measure, as well as to aid the design of provision to support wellbeing.

### Valuing physical activity for wellbeing

Definitions of wellbeing have predominantly focused on psychological, social and emotional aspects despite evidence that physical health and activity is important and intertwined with wellbeing in healthy adults [20] and young people [21]. Understanding engagement and access to physical activity for supporting physical health has important ramifications for subjective and psychological wellbeing, as low levels of physical activity are situated alongside low levels of social, emotional and mental wellbeing [1]. Research has consistently shown the benefits of physical health and activity for a range of mental health and wellbeing areas. For example, a systematic review and meta-analysis reported increased physical activity has been shown to have the potential to reduce anxiety and depressive symptoms and improve self-esteem in children and youth [22]. A UK school case study report found mean wellbeing scores were higher for pupils who engage in more days of physical activity a week, and ratings of physical health was the largest unique predictor of wellbeing [23]. Sports England reported, using large-scale survey data, a positive association between increased levels of sport and reduced feelings of loneliness, as more active children and young people are less likely to “often or always” feel lonely [9]. These results highlight that physical activity is a mechanism for supporting wellbeing and mental health [24]. Therefore, social, emotional and physical domains are identified as relevant characteristics and adopted in the present study [25].

There are biological, physical and social mechanisms for the proposed benefits of physical activity for wellbeing. Causal research suggests increased physical activity leads to increased endorphins, dopamine, serotonin, and noradrenaline concentrations that are related to reduced symptoms associated with anxiety and depression and increased moods protective of mental health conditions [20]. Increased physical activity can lead to better physical self-perceptions in terms of appearance, fitness and ability [18, 20]. Socially, physical activity has the potential to improve feeing of connectedness and belonging, as physical activity often takes place in social group and teams, enabling the building of connections and relationships with others, and thus supporting social wellbeing [17, 19].

Latent class analysis has gained popularity for exploring latent class profiles for mental health and wellbeing [7]. However, links across physical wellbeing, health and physical activity are under explored given the known associations, moreover there is a clear lack of work understanding these concepts and associations in children and young people. A large scale Spanish study did establish profiles of mental wellbeing in relation to physical activity practice, and results showed that adult individuals, aged 16 to 88 years, in the high wellbeing group showed significantly higher physical activity patterns than those in moderate and low wellbeing groups [26]. However, another study with Canadian medical students aged 19 to 42 years, found those in the high mental health profile engaged in more mild physical activity compared to those in the moderate and low profiles [27]. Such insights suggest it may not necessarily be the frequency and intensity of physical activity that is important for mental health; rather the type and form, and moderate physical activity tends to be social and promote fun. In these instances, it may be social mechanisms that are underlying the benefits of such activity. These results indicate the relationship between physical activity and wellbeing is complex and requires a considered approach. This study first aims to explore the role of physical wellbeing alongside subjective and psychological wellbeing to determine how they co-exist by using data driven profiles of physical, subjective and psychological wellbeing in adolescents.

### Valuing physical activity and wellbeing in education

Physical education (PE), school sport and physical activity are important components of the national teaching curriculum in the UK. Within the UK there is a recommendation of two hours of timetabled PE a week, which is considered to benefit school attainment, wellbeing and personal and social skills and development [28]. There is evidence that physical activity and wellbeing is associated with a variety of educational domains, including attainment and school engagement, and such physical activity can aid general cognitive functioning including memory, motor and perceptual skills, and IQ [29]. Physical wellbeing and activity can also benefit emotional literacy [48], again, mechanisms tied to this purport that the social benefits of physical activity build social and emotional skills such as learning to manage and respond to emotions of oneself and others [30].

There is also a need to understand who is at greatest risk of reduced physical activity and thus those at need for targeted provision and support within education settings. There is work noting demographics trends for engagement in physical activity. For instance, least likely to be active are older students (ages 15–16), girls, children and young people from less affluent families, and children and young people of Black, Asian and Other ethnicities [8, 44–46]. Physical activity levels are associated with low levels of social, emotional, and mental wellbeing and exacerbated by increased inequalities [1]. Thus, understanding context is vital to recognise the challenges for education provision and those most at need. Yet these factors have not been studied simultaneously within adolescents and in relation to data driven profiles of wellbeing across multiple domains. Therefore, a second aim was to understand what differentiates these profiles, and understand what role do physical activity levels, education variables and student demographics play in differentiating between these groups.

### The current study

The current study collected self-reported data from adolescents at secondary schools in the England and Wales, whose self-reported physical wellbeing, subjective and psychological wellbeing fuelled the exploration of different wellbeing profiles. A further set of aims was to explore how the composition of these profiles differed by demographic, physical activity and educational characteristics. The research questions were as follows:

1. What are the different profiles of physical, subjective and psychological wellbeing within young people?
2. How do demographic characteristics of age, speaking English as an additional language (EAL) and perceived socio-economic status (SES) relate to profile grouping.
3. How does physical activity measures including the number of days of physical activity a week, amount of time spent exercising out of school a week, and amount of time sedentary a week relate to ability relate to profile assignment?
4. How do education variables emotional literacy (differentiating emotions, verbal sharing of emotions, not hiding emotions, bodily awareness of emotions, attention to others’ emotions, and analyses of emotions), school belonging, and perceptions of learning ability relate to profile assignment?

## Method

### Participants

The sample included 366 pupils aged 11-16yrs (mean age 12.75) from three mainstream secondary schools across England. The majority of the sample were born in England (72.9%) and approximately a fifth (21.7%) spoke English as an additional language and this is comparable to national trends in English schools [31]. Perceived family economic status was measured by asking pupils to self-report on a scale how well off they felt their family was, this is a common tool used in national surveys such as the Health Behaviour in School-Aged Children surveys [32], pupils responded on a Likert scale of 1–5 (not well of at all, not so well off, average, quite well off, very well off). Just under half the sample described their families as of average economic status. Sample characteristics are shown in Table 1.

**Table 1:** Sample characteristics

		Mean (range)
Mean age in years		12.75 (11-16yrs)
		**n (%)**
Gender	Girls	170 (46.6%)
	Boys	180 (49.3%)
	Other	6 (1.6%)
	Prefer not to say	9 (2.5%)
Born in England	Yes	266 (72.9%)
	No	92 (24.1%)
Speak English as an additional language	English	287 (78.4%)
	Other	77 (21.2%)
Perceived family economic status	Very well off	28 (7.8%)
	Quite well off	78 (21.7%)
	Average	163 (45.4%)
	Not so well off	14 (3.8%)
	Not well of at all	7 (19%)
	Prefer not to day	21 (5.7%)
	Unsure	48 (13.4%)

### Measures

#### Physical wellbeing – KIDSCREEN-27

The physical wellbeing (7 items) subscale of the child self-report version of KIDSCREEN-27 [33] was used for use with children age 8 to 18 years. The child reads a statement indicating their level of agreement on a five-point scale (e.g., never, one day, some days, most days, every day). The KS27 is psychometrically robust, with high internal consistency (> 0.8), good reproducibility (> 0.6) and criterion validity [33]. Scale reliability in the current study was Cronbach’s Alpha of 0.83.

#### The stirling children’s wellbeing scale (SCWBS)

The SCWBS [16] is a holistic measure of subjective and psychological wellbeing, it consists of 12 items measuring subjective (Positive Emotional State, 6 items) and psychological wellbeing (Positive Outlook, 6 items) in children 8 to 15 years of age. All items on the scale are rated on a 5-point Likert-based scale (never, not much of the time, some of the time, quite a lot of the time, all of the time). Scale reliability is strong, scoring above 0.8 [16]. Scale reliability in the current study was a Cronbach’s Alpha of 0.88 for positive emotional state and 0.77 for positive outlook.

#### Physical activity

Three questions asked about the frequency of physical activity and adopted framing consistent with existing self-reports sought from children (e.g., Health Behaviour in School-Aged Children national surveys). Physical activity was defined as any activity that increases heart rate and makes you get out of breath some of the time. Physical activity could have been part of sports, school activities, playing with friends or walking to school. Some examples offered within the survey were running, rollerblading, biking, dancing, swimming, and football. Students were asked: (1) In the past week, on how many days have you taken part in 60 min or more of physical activity that makes you feel warmer and makes your heart beat faster? It does not have to be 60 min in one go, you can add together different bits of activity you do in one day (2) Outside school hours: how many hours a week do you usually exercise in your free time so much that you get out of breath or sweat? and (3) On an average school day, how many hours do you spend in front of a TV, smart phone, computer, tablet or similar electronic device when you watch shows, videos, play games, use social media. Do not count time on schoolwork for this time.

#### Emotional literacy - emotion awareness questionnaire (EAQ)

The Emotion Awareness Questionnaire (EAQ) [34] is a 30-item scale measuring emotional literacy skills in children in 8–16 years of age. Six sub-scales address emotional awareness: differentiating emotions, verbal sharing of emotions, bodily awareness of emotions, acting out emotions, analyses of emotions and attention to others’ emotions. Children respond to each statement rating how true each item is for them on a 3-point scale (1 = not true, 2 = sometimes true, 3 = often true). Item reliability is reported between 0.64 and 0.77 [34, 35]. Scale reliability in the current study ranged between a Cronbach’s Alpha of 0.65 (attention to others’ emotions) to 0.84 (differentiating emotions).

#### School engagement - the psychological sense of school membership scale (PSSM)

The Psychological Sense of School Membership Scale (PSSM) [36] was used to measure engagement and connections in the school environment for pupils aged 10 and over. The scale consists of 18 items measured using a Likert scale of 1 (not true at all) to 5 (completely true). The scale has high reliability of over 0.8, construct validity and relationships with a variety of educational outcomes [36]. Scale reliability in the current study was Cronbach’s Alpha of 0.73.

#### Learning/academics - myself as a learner (MALS)

The Myself as a Learne ler (MALS) [37] is designed to assess children’s perceptions of their learning abilities, in pupils 8–16 years of age. The scale is a 20-item measure that uses a Likert scale from 1 (yes definitely) to 5 (definitely not) and provides brief items. The scale has a maximum score of 100 and minimum score of 20. The scale shows good reliability at 0.85 [37] and is predictive of academic achievement [38]. Scale reliability in the current study was Cronbach’s Alpha of 0.93.

### Procedure

This data comes from a larger project funded by the Youth Sport Trust exploring wellbeing provision in schools [19]. Ten schools were recruited by the Youth Sport Trust representing diversity in settings (mainstream primary and secondary schools and alternative provision special schools), geographical areas of the UK (England, Scotland and Wales), variation in school demographics such as proportion of children eligible for free school meals (FSM), special educational needs and/or disabilities (SEND) and English as an additional language (EAL). These project reports of data from the four recruited secondary school in England and Wales. Schools were invited to complete online pupil surveys with a selection of their pupils (up to 150 per school) to support feasibility and practicality, while maintaining a range of age groups and abilities. For example, we suggested inviting pupil in Year Groups 7 (ages 11–12) and 9 (ages 13–14) in secondary schools, we selected Year 7 who have made the transition from primary education and Year 9 who will be making the transition to examination courses. This pragmatic approach reduced the burden on participating schools.

We ensured that participants were clearly informed at all stages. Information and consent letters were created and sent to schools to distribute to parents/carers. Pupil friendly and age-appropriate information and assent letters were also created. Opt-out parent/carer consent procedures were adopted for pupil online surveys (as there was no direct contact with pupils who completed the surveys anonymously and no personal data was collected).

Surveys were completed online (although paper copies were available, this request was not taken up). School staff administered the survey with groups of pupils at a time convenient for them, this tended to be in form time. Teachers were provided with crib sheets and step-by-step instructions on how to access and introduce the survey to their pupils.

### Data analysis

Latent profile analysis (LPA) uses a person-centred approach to classify individuals from a heterogeneous population into homogenous subgroups [39]. In LPA, categorical latent variables are formed that distinguish groups with different constellations of the observed values. The appropriate number of groups is decided based on an evaluation of a series of model fit indices. Continuous predictors of emotional state, positive outlook, and physical wellbeing were used to create wellbeing profiles. Analyses were conducted in Mplus v8.4 [40]. Two cases were missing data on all latent profile predictors positive emotional state, positive outlook, and physical wellbeing and were discounted from the analysis. Further missing data was determined as missing at random (MAR) based on observations that there were systematic differences between the missing and observed values on certain variables, but was largely minimal (e.g., under 5% for demographic variables of age, gender and EAL and physical activity variables, while 8% was missing for both emotional literacy and psychological belonging, however 20% missing for perceived SES and 26% missing for perceptions of learning ability). Therefore, to make use of both fully and partially observed cases and reduce the bias associated with attrition full information maximum likelihood (FIML) was used. FIML is recommended as an efficient method for handling missing data in latent class analysis [37] and has been shown to produce unbiased parameter estimates and standard errors under MAR conditions [41] as be shown to be an efficient method for handling missing data for modelling algorithms such as LPA. Data across all available variables were used in the FIML to help impute missing data. A copy of the Mplus syntax and reporting specific model parameters used can be found in the supplementary information.

#### Latent profile analysis

Best practice guidance for LPA was followed [42]. Exploratory LPA was used to explore the number and make-up of different subjective physical and psychological wellbeing profiles, therefore, allowing the identification of different profiles and the characterised that were associated with membership [39]. This data driven exploratory approach was selected due to the dearth of research investigating how physical and psychological wellbeing simultaneously inform subjective wellbeing. Three continuous variables were used to explore profiles, positive emotional state and positive outlook (both subjective and psychological wellbeing from the Stirling Children’s Wellbeing Scale), and physical wellbeing (from the KIDSCREEN-27). The scores across these three variables were measured on different scales so to allow them to be compared and interpreted values were transformed to standardised z-scores [42].

The analysis followed the recommended six steps for LPA [42]. Step one was to clean the data and check distributions and statistical assumptions such as mean scores and data distributions and comparisons with national benchmark data. Step two used an iterative model building approach, running model by model from one profile upwards to explore model fit and changes at each stage until convergence was reached. Step three was to report and explore model fit indices to determine model fit and interpretability. Reported indices included, loglikelihood (LL), Akaike Information Criterion (AIC), Bayesian Information Criterion (BIC), and sample-size adjusted Bayesian Information Criterion (ssa BIC), and as values across these indices decreased a better model fit was demonstrated. Significant Lo-Mendell Rubin Adjusted Likelihood Ratio Test p value (LMR-LRT *p*) indicates a significant change in the model and better model fit at each stage. Class sizes and proportions were recorded at each stage to display the number of assigned members per group. Entropy values above 0.80 indicated acceptable classification accuracy [43]. As per best practice, this value was examined but not used for selecting the best model [43, 44].

Step four involved examining patterns of profiles and model fit values were reviewed to support understanding the best profile fit. Class sizes and proportions was also used to support determining theoretically meaningful group sizes; models with low membership (e.g., 5%) were discounted as the profile could be unstable and models with small class sizes are less likely to be generalisable than models with fewer, well-distributed, classes [45]. Mean z-scores for profile membership predictors (physical wellbeing, positive emotional state, and positive outlook) were reported graphically and compared to interpret profile membership.

Step five moved to interpret profiles in terms of their characteristics relating to demographics, physical activity and educational variables to support convergence of the model. This was computed three times adding additional covariates at each step: (1) Demographic variables (e.g., age, gender, EAL, and perceived SES). (2) physical activity averaged per week (the number of days of physical activity, volume of time spent exercising out of school and volume of sedentary time). (3) Education variables (e.g., emotional literacy domains of differentiating emotions, verbal sharing of emotions, not hiding emotions, bodily awareness of emotions, attention to others’ emotions, and analyses of emotions (EAQ subscales), and school belonging (PSSM) and perceptions of learning ability (MALS)). As recommended and as entropy was just under 0.8, the ML three-step approach was used to investigate the relationship between covariates and profile membership while accounting for classification uncertainty [46]. This involved selecting the optimal latent profile solution, saving the measurement parameters and classification error and estimating the relationship between covariates and latent profile membership while accounting for classification error.

Step six presents the latent profile solution alongside a narrative of the process and outcomes with accompanying tables of model fit statistics and figures illustrating profile membership on each of the predictor variables of physical wellbeing, positive emotional state and positive outlook.

## Results

Mean scores for physical, subjective and psychological welling, physical activity and educational variables are shown in Table 2, and data was reasonably normally distributed [47]. Subjective and psychological wellbeing were lower than previously reported averages of approximately 42–44, in two UK samples using the SCWBS with children aged 8 to 15 years in 2015 and 9 to 12 years in 2018 [16, 48]. However, this data was collected in the aftermath of the COVID-19 pandemic (November to December 2022) while associated social restrictions were beginning to ease and such context ought to be recognised as likely influencing these scores. As children are entering adolescence and early adulthood it is a time of increasing pressure where school expectations are changing, transitional periods and pressures are encountered and peer and social pressures experienced by adolescents disrupt wellbeing and can lead to variations in such scores [5]. Further, the sample were secondary school-aged and such age trends for lower wellbeing are congruent with other data for adolescence [16, 31].

On average pupils reported they spend 3.5 days a week taking part in 60 min or more of physical activity. Outside of school, 53.7% of students spent up to one hour a week, and 25.6% spent up to 2–3 h a week taking part in exercise in their free time. On an average school day, 52.2% of students reported spending over three hours in front of a TV, smart phone, computer, tablet or similar electronic device when watching shows or videos, playing games or using social media, and 36.9% up to 2–3 h a week. Mean scores and ranges for emotional awareness were in line with published data from adolescents collected during the COVID-19 pandemic [49], scores for school belonging were comparable with findings in published (albeit dated) research with a similar population [36], and scores leaned to positive learning perceptions and aligned with established data [38].

**Table 2:** Descriptive statistics for psychological and physical wellbeing, emotional literacy, school belonging and perceived learning ability

Variable (possible score range)		Mean (SD)
Physical wellbeing (5–25)		16.91 (3.92)
Psychological wellbeing	Total (12–60)	38.64 (8.44)
	Positive Emotional State (6–30)	19.05 (4.83)
	Positive outlook (6–30)	19.53 (4.11)
Emotional literacy	Differentiating emotions (7–21)	15.01 (3.79)
	Verbal sharing of emotions (3–9)	5.56 (1.78)
	Not hiding emotions (5–15)	8.63 (2.65)
	Bodily awareness of emotions (5–15)	9.96 (2.66)
	Attention to others’ emotions (5–15)	12.96 (1.78)
	Analyses of emotions (5–15)	10.09 (2.56)
Sense of School Membership		54.36 (8.99)
Myself as a learner		64.25 (14.28)
No. days of > 60 min of physical activity (0–7)		3.55 (1.87)
		**n (%)**
Amount of exercise out of school	Low (0–1 h)	191 (53.7%)
	Moderate (2–3 h)	91 (25.6%)
	High (3 or more hours)	74 (20.8%)
Amount of time sedentary a week	Low (0–1 h)	31 (8.5%)
	Moderate (2–3 h)	135 (36.9%)
	High (3 or more hours)	191 (52.2%)

### Wellbeing latent profile analysis

In an LPA, a categorical latent variable is formed that distinguishes groups with different constellations of the observed values. The appropriate number of groups is decided via fit indices such as the BIC. Latent profile analysis was carried out iteratively from model 1 with a one profile solution, reaching convergence with entropy stabilising at 0.81 at model 5 identifying five profiles (Table 3). Interpretation of a series of fit indices can help to approximate the correct number of profiles. Comparing the values of the BIC across the models showed a decrease as more profiles were created, indicating better model fit with an increasing number of profiles. The LMR-ALRT showed a significant improvement in model fit from model 2 to model 3 (*p* = .038) but after that improvements are non-significant. Therefore, model fit indices improved as more models are created but stabilised at around model 3 suggesting a three-profile model was optimal. The entropy value for the three-profile model was 0.76 which is around the cut-off of 0.8 for acceptable classification quality. In addition, the profiles of model 3 made theoretical sense and differentiated three levels of wellbeing.

**Fig. 1:**
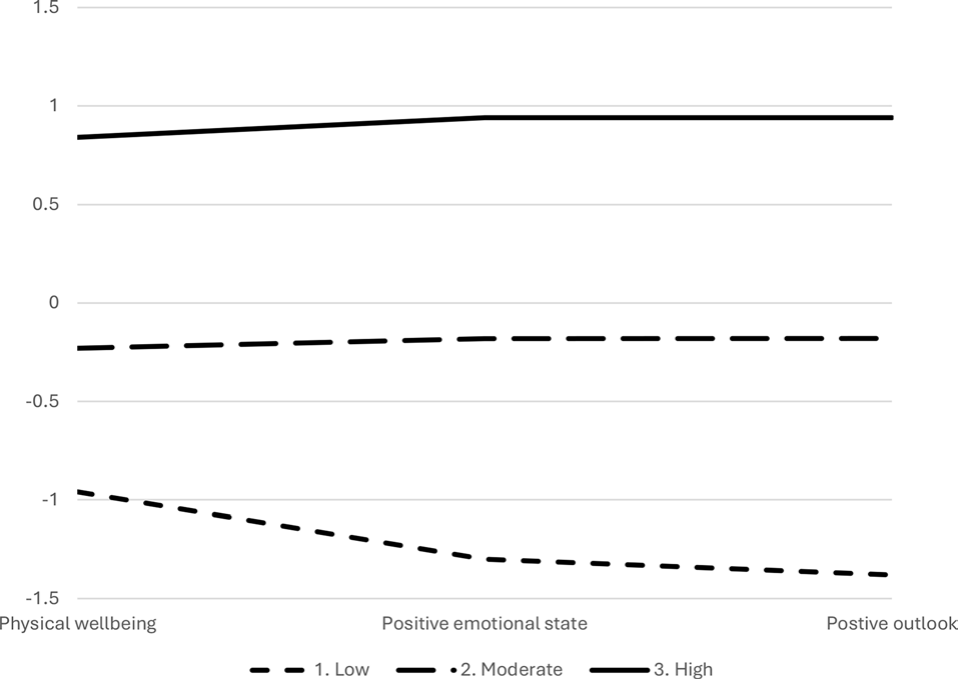
Mean standardised scores across profile predictors positive emotional state, positive outlook and physical wellbeing for a 3-profile solution

**Fig. 2:**
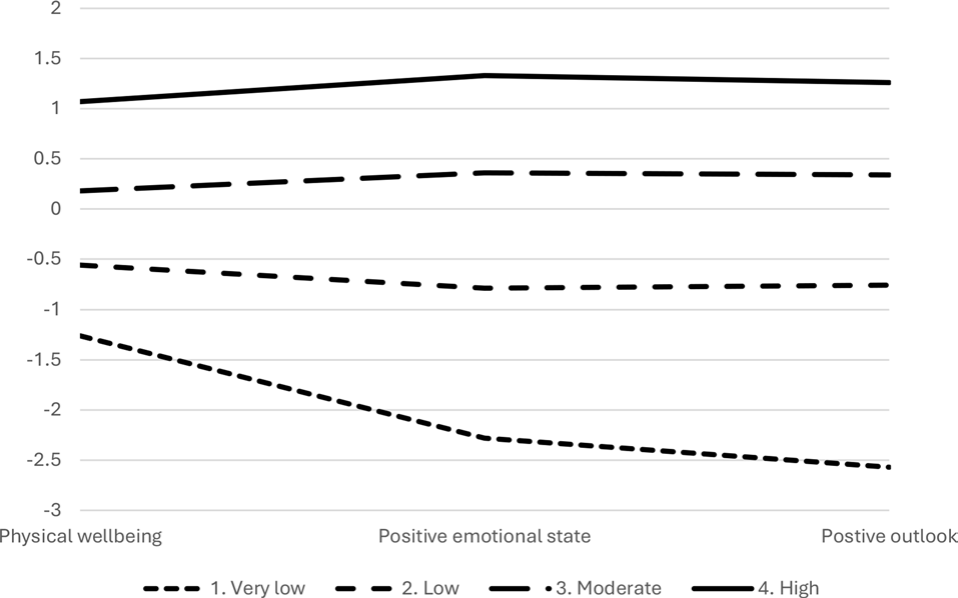
Mean standardised scores across profile predictors positive emotional state, positive outlook and physical wellbeing for a 4-profile solution

**Fig. 3:**
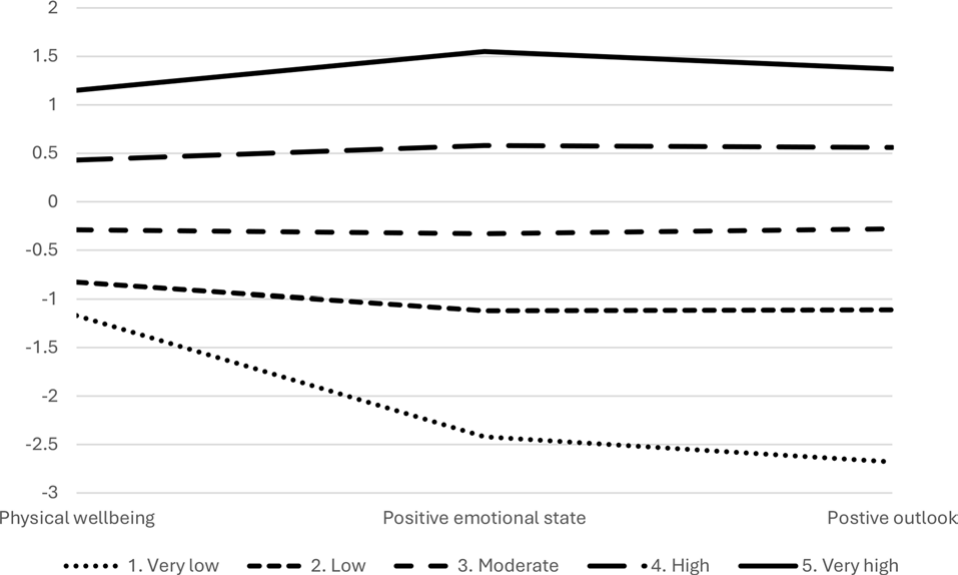
Mean standardised scores across profile predictors positive emotional state, positive outlook and physical wellbeing for a 5-profile solution

The three-profile model consisted of profile 1 – low wellbeing (*n* = 68, 19%) displaying low scores across positive emotional state, positive outlook and physical wellbeing, 2 – moderate wellbeing (*n* = 168, 46%) characterised by average levels across positive emotional state, positive outlook and physical wellbeing, and 3 – high wellbeing (*n* = 128, 35%) showing high scores across positive emotional state, positive outlook and physical wellbeing (Fig. 1). The four-profile model showed an increase in entropy to 0.81, but there was no suggestion of a significantly better fit (*p* = .091). Although the BIC, AIC, sssBIC continue to decrease after the three-profile solution, indicating better fit, this is quite common, the improvement in fit is minimal as indicated by the LMR-LRT that suggests no significant improvement in fit. However, it had extracted a fourth profile with very low wellbeing (Fig. 2) and characterised specifically by much lower wellbeing for positive emotional state and positive outlook, but not a similar level of reduced physical wellbeing. This group of 10 members was deemed too small to support generalisation or further analysis of meaningful profile membership [42]. The five-profile model stabilised with entropy remaining at 0.81, there was no suggestion of a significantly better fit (*p* = .078). This set of profiles also illustrated a small very low wellbeing group consisting of eight members, separate to a moderate wellbeing group (Fig. 3).

**Table 3:** Profile fit indices and profile sizes for each model

Model	LL	AIC	BIC	ssa BIC	Entropy	LMR-ALRT *P*	Profile sizes *n* (proportion)
1	-1498.32	3008.63	3032.01	3012.98	-	-	364 (1.0)
2	-1340.81	2701.61	2740.58	2708.86	0.77	< 0.001	181.53 (0.50), 182.47 (0.50%)
**3**	**-1289.39**	**2606.77**	**2661.33**	**2616.92**	**0.76**	**0.038**	**68.10 (0.19)**,** 167.66 (0.46)**,** 128.25 (0.35)**
4	-1256.35	2548.70	2618.85	2561.75	0.81	0.091	10.14 (0.03), 140.58 (0.39), 150.85 (0.41), 62.43 (0.17)
5	-1232.83	2509.56	2595.40	2525.60	0.81	0.078	8.51 (0.02), 71.20 (0.20), 113.55 (0.31), 43.17 (0.12), 127.55 (0.35)

Note: LL – Loglikelihood, AIC – Akaike Information Criterion, BIC - Bayesian Information Criterion, ssa BIC - sample-size adjusted Bayesian Information Criterion, LMR-ALRT p - Lo-Mendell Rubin Adjusted Likelihood Ratio Test p value. Bold text indicates models selected for subsequent analysis

### Characterising profile assignment

As the three-profile solution suggested the overall best fit, further analysis used the three-profile solution to understand profile composition and the characteristics that differed between the three profiles. Three covariate models were run adding the series of demographic, physical activity and educational covariates to determine significant variables associated with each profile. Profile 3 (high wellbeing) was used as the reference group, allowing comparisons with moderate and low wellbeing groups. Models were separately computed for demographic, physical activity and educational covariates to support convergence of the model and are summarised in Table 4. Results found demographic covariates age, gender and perceived SES differed between profiles. Older students were more likely to be members of profile one (low wellbeing) and profile two (moderate wellbeing) than profile three (*B* = 0.37, SE = 0.18, *p* = .039 and *B* = 0.56, SE = 0.17, *p* < .001). Boys were *less likely* to be members of the low and moderate wellbeing profiles than the high wellbeing profile (*OR* = 0.42, *B* = -1.96, SE = 0.45, *p* < .001 and *OR* = 0.43, *B* = -0.85, SE = 0.35, *p* = .013). Students reporting lower SES was more likely to be members of the low and moderate wellbeing profiles than the high wellbeing profile (*B* = -0.74, SE = 0.31, *p* = .017 and *B* = -0.79, SE = 0.23, *p* = .002). Speaking EAL did not differ between group profiles (*OR* = 1.08, *B* = 0.08, SE = 0.49, *p* = .88 and *OR* = 1.30, *B* = 0.26, SE = 0.43, *p* = .542).

For physical activity covariates, students who reported engaging in fewer hours of physical exercise a week were more likely to be members of the low and moderate wellbeing groups (*B* = -0.62, SE = 0.20, *p* = .002 and *B* = -0.31, SE = 0.10, *p* = .003). Students who engaged in moderate levels of sedentary activity compared to high were less likely to be in the low or moderate wellbeing groups (*OR* = 0.35, *B* = -1.05, SE = 0.46, *p* = .022 and *OR* = 0.37, *B* = -1.00, SE = 0.34, *p* = .003) - this suggests a moderate amount (rather than high or low amounts) of sedentary activity is potentially better for wellbeing.

Educational covariates differed between profile membership. The low wellbeing group was characterised by significant lower emotional literacy, and lower scores for differentiating emotions, not hiding their emotions, bodily awareness of emotions and analysing emotions was associated (*B* = -0.33, SE = 0.14, *p* = .019, *B* = -0.45, SE = 0.20, *p* = .024, *B* = -0.53, SE = 0.18, *p* = .003 and *B* = -0.36, SE = 0.18, *p* = .046), and significantly lower feelings of school belonginess (*B* = -0.20, SE = 0.08, *p* = .009) and lower perceptions of learning ability (*B* = -0.13, SE = 0.04, *p* < .001). The moderate wellbeing group was characterised by significantly lower emotional literacy for bodily awareness of emotions significantly differed (*B* = -0.29, SE = 0.14, *p* = .033), significantly lower feelings of school belonginess (*B* = -0.18, SE = 0.06, *p* = .002) and lower perceptions of learning ability (*B* = -0.08, SE = 0.03, *p* = .009).

**Table 4:** Co-variate analyses results for associations for three-profile solution

Co-variate				Profile 1 – low wellbeing	Profile 2 – moderate wellbeing
				***Beta co-efficient (SE)***	***Beta co-efficient (SE)***
Demographic	Age			0.37 (0.18)*	0.56 (0.17)***
	Gender (compared to girls)	Boys		-1.96 (0.45)***	-0.85 (0.35)*
		‘Other’ gender		66.23 (95.56)	65.07 (0.35)
	Speaking EAL			0.08 (0.49)	0.26 (0.43)
	Perceived SES			-0.74 (0.31)*	-0.79 (0.26)**
Physical activity	The number of days of physical activity a week			-0.62 (0.20)**	-0.31 (0.10)**
	Amount of time spent exercising out of school a week (compared to high)		Low	0.65 (0.75)	0.33 (0.43)
			Moderate	0.48 (0.75)	0.66 (0.42)
	Amount of time sedentary a week (compared to high)		Low	-0.74 (0.65)	-1.33 (0.71)
			Moderate	-1.05 (0.46)*	-1.00 (0.34)**
Educational	Emotional literacy	Differentiating emotions		-0.33 (0.14)*	-0.10 (0.09)
		Verbal sharing of emotions		-0.39 (0.25)	-0.35 (0.18)
		Not hiding emotions		-0.45 (0.20)*	-0.23 (0.12)
		Bodily awareness of emotions		-0.53 (0.18)**	-0.29 (0.14)*
		Attention to others’ emotions		-0.16 (0.19)	-0.16 (0.14)
		Analyses of emotions		-0.36 (0.18)*	-0.02 (0.10)
	School belonging			0.20 (0.08)**	-0.18 (0.06)**
	Perceptions of learning ability			-0.13 (0.04)***	-0.80 (0.03)**

Note: Profile 3 (high wellbeing) is used as the reference group

* *p* < .05, ** *p* < .01, *** *p* < .001

## Discussion

This study used LPA to identify data driven profiles of physical, subjective and psychological wellbeing. The results identified three profiles, low wellbeing (displaying low scores across physical wellbeing, positive emotional state and positive outlook), moderate wellbeing (characterised by average levels across physical wellbeing, positive emotional state and positive outlook), and high wellbeing (showing high score across physical wellbeing, positive emotional state and positive outlook). Lower wellbeing was associated with demographic variables such as being older, being a girl, lower perceived socio-economic status, fewer hours of physical activity a week, and education variables of lower emotional literacy, school belonging and perceptions of learning.

The first research question aimed to generate different profiles of wellbeing. These three profiles identified similar patterns of physical, subjective and psychological wellbeing for the low, moderate and higher wellbeing profiles. This emphasises the intertwined relationship across these areas of wellbeing [20, 21], as we did not find for example, a profile with high subjective and psychological wellbeing but low physical wellbeing and vice versa. While three profiles were statistically identified, the data does suggest the possibility of further groups of small numbers of very low wellbeing (referred to as profile four and five earlier). While these numbers were too small to examine with meaningful profile analysis, this does not discount clinical groups emerging that warrant further exploration and understanding, as here we may have identified adolescent groups that could lead to later clinical diagnosis and health concerns into adulthood. Further research with larger numbers may wish to explore the stability of more extreme low scoring risk groups that will likely be a smaller proportion of any data set, yet have sufficient numbers to represent and understand such an important group who may be the most vulnerable [42]. So, while there was evidence of a more extreme drop in subjective and psychological wellbeing compared to physical wellbeing, this pattern is difficult to generalise from because of small group numbers in these higher risk groups. A potential reason may be protective factors such as youth, this is a population of adolescents with a mean age 13, who are younger and in general good physical health at this age. It may also be indicative of a lag in reductions of physical health and wellbeing, behind subjective and psychological wellbeing, and a notion that mental health problems predict later physical health concerns in adolescents [50]. A second possibility for the difference in physical wellbeing compared to subjective and psychological wellbeing in the very low profiles could be the protective nature of schools, with physical education (PE) and sport curriculums offering a protective factor of regular engagement in physical activity [21]. Indeed students on average spent 4.5 days a week taking part in 60 min or more of physical activity and outside of school nearly half of pupils typically spent 2 h or more a week taking part in exercise, these values suggest the a good proportion of the sample were meeting the Chief Medical Officers’ guidelines of taking part in sport and physical activity for an average of 60 min or more everyday [3]. Whether these patterns of wellbeing profiles would remain stable over time and continue to be observed in adult populations would be worthy of consideration.

The self-reported subjective and psychological wellbeing of adolescents in the current study was lower than previously published benchmarks, for example the mean score within the study was reported at 39 and is lower than scores reported in previous studies of for example 42 [48]. Data for the study was collected during the end of the COVID-19 pandemic and as social restrictions had recently been eased and a return to school started. It is not surprising therefore that scores for wellbeing were somewhat lower during this time of uncertainty and challenge and is in line with corroborating evidence [1] that wellbeing was lower in 2022 compared to 2021, and substantially lower than in 2020. Such evidence indicates the enduring effects of the pandemic on children and young people’s wellbeing. This trend for a dip in wellbeing post pandemic is echoed globally [51] with the World Health Organization reporting at 25% increase in prevalence of anxiety and depression worldwide, and with a disproportionately higher effect for young people [52]. Further, the lower scores detected in the study could reflect the age of student sample as there are notable trends for lower wellbeing in older children [1, 16]. As children are entering adolescence and early adulthood it is a time of increasing pressure. School expectations are changing and examinations pressures increasing, transitional periods and pressures are encountered as physical and biological changes occurring, and peer and social pressures are experienced such as fitting in with peers and romantic relationships that can all impact wellbeing [6].

Research question two aimed to explore how wellbeing profile groups were associated with demographics characteristics. Profiles followed expected trends concerning age, gender and SES. The transition into adolescence marks a time of change physically, socially, emotionally and psychologically that brings new stresses, concerns and sensitivities, as thus can leads to reduced wellbeing compared to childhood [8]. Such periods of transition are reported to impact girls more than boys [53], which would explain that girls were more likely to be members of the moderate and low wellbeing profiles than boys. These trends mirror previous research findings regarding age and gender differences in wellbeing and mental health [54]. Adolescents reporting perceived lower perceived SES status were likely to be member of moderate and low wellbeing profile, again mirroring trends in the general population [55] as well as adolescents specifically [56].

Research question three explored how levels of physical activity differentiated wellbeing profiles. The mean wellbeing scores were higher for pupils who engaged in more days of physical activity a week and ratings of physical health was a large unique predictor of wellbeing. This result highlights the potential positive effects of increased physical activity on wellbeing. Increased physical activity is a suggested mechanism for supporting wellbeing and mental health [24] and supports a notion that school physical activity interventions may improve wellbeing children and young people [21] through the promotion of skills such as resilience and social relationships. Thus, the benefits of a school curriculum that incorporates a consistent focus on physical activity may see an impact on wider student outcomes. However, it is also possible that children with superior wellbeing are more likely to engage in physical activity. An interesting finding regarding levels of sedentary activity suggested a moderate amount rather than high or low amounts of sedentary activity is better for wellbeing. Therefore, while engaging in psychical activity can be beneficial for wellbeing, so can *moderate levels of sedentary activity* that encourages time to relax and engage in fun activities such as watching TV, spending time on digital activities such as games and social media [24].

Finally, membership of the wellbeing profiles was differentiated by education variables. The moderate and low wellbeing groups was associated with education related variables of lower emotional literacy, school belonging and perceptions of learning. These trends were expected, as lower wellbeing is thought to impact and be impacted by a host of school-related variables. Students better able to identify, understand and manage emotions, are better placed to manage decisions and interpersonal situations constructively, and less likely to experience emotional distress and conduct problems [57]. Less inclusive and engaging school environments have been shown to create feelings of loneliness that predict mental health problems and lower subjective wellbeing [58]. Bidirectional relationships between wellbeing and achievement are found, suggesting that higher academic achievement leads to higher wellbeing, higher academic achievement and perception of learning positively predicts wellbeing and positive outlook [59].

### Generalisability

The role of physical activity and wellbeing have been identified in three profiles that used a dataset to understand characteristics influencing wellbeing within schools. It should be recognised that differences between profiles were largely quantitative in nature, so rather than distinguishing profiles based on differences across wellbeing domains, profiles identified wellbeing levels. Including physical wellbeing as part of determining levels could be preferable in future research. However, while the dataset may appear small it is within recommended numbers for this type of analysis [39, 42]. Further research with larger numbers is warranted to consider the possible fourth and fifth profile groups identifying small but important membership to very high-risk groups and allow a closer consideration of profile characteristics and variation across different wellbeing domains. Generalisation and transferability of these results could be treated somewhat cautiously. The data was collected post-pandemic as social restrictions were easing. Across this period reduced wellbeing and physical activity was acknowledged for the adolescent populations nationally and internationally [31, 52]. Thus, there is a basis for revisiting these profiles and looking at the relationship between physical activity and wellbeing again and potential over a longer period as the post-pandemic period may have affected the nature of the profiles captured within the data. A next step would be to understand profiles longitudinally as well as consider the direction of factors associated with wellbeing profile membership. For example, is physical activity a protective factor for subjective and psychological wellbeing, or is it that those with higher levels of wellbeing engage in more physical activity? Similarly, greater understanding of how profiles across physical, subjective and psychological wellbeing change and diverge as we age is warranted. A further possibility is research that considers a wider range of age groups – fuelling lines of investigation that target variation between younger and older children and goes on to examine whether different forms and types of physical activity provision fuels differential impacts of wellbeing.

## Conclusion

Understanding broader profiles of wellbeing that encompass physical health and wellbeing can help identify young people at risk and inform how educational institutions can promote mental health for students via school provision. Exploring the role of physical health and activity for wellbeing offers an opportunity to understand the potential role physical wellbeing can play for adolescent mental health. It also offers an understanding of possible preventive measures and factors associated with promoting wellbeing in educational settings. Increased physical activity can also operate as a protective factor and provide the potential to reduce symptoms of depression and anxiety in adolescents [60], and foster resilience [61]. Schools that endorse physical activity and support physical literacy (the positive attitudes towards physical activity) may improve the wellbeing of children and young people [21] through the promotion of skills such as resilience and social relationships.

## Electronic supplementary material

Below is the link to the electronic supplementary material.

Supplementary Material 1

## Data Availability

The datasets generated and analysed during the current study are not publicly available due to ethical restrictions.
