# Supplementary material for: Exploring physical, subjective and psychological wellbeing profile membership in adolescents: a latent profile analysis
Source: BMC Psychol. 2024 Dec 4;12:720. doi: 10.1186/s40359-024-02196-5 (PMC11619419; doi:10.1186/s40359-024-02196-5)
Supplement: Supplementary file 1 — Supplementary Material 1 [file 40359_2024_2196_MOESM1_ESM.docx]

**Appendix**

***A. Mplus syntax to generate profiles***

VARIABLE:

NAMES = ID SCH AGE GENDER GIRL BOY OTHERGEN ENG LANG SEND

SES WB WBEM WBOUT PHWB DIFFEM VERBEM HIDE BODY ATT ANA

SB MALS PADAY PHAct2 PHACTHR PAHRLO PAHRMOD PAHRHI

PHAct3 PASEDHR PASEDLO PASEDMOD PASEDHI ZWBEM

ZWBOUT ZPHWB;

USEVARIABLES = ZWBEM ZWBOUT ZPHWB;

MISSING = ALL (-99);

CLASSES = C(1); ! increase number to extract number of exploratory profiles

ANALYSIS:

TYPE = MIXTURE;

STARTS = 1000 250;

STITERATIONS = 50;

PROCESSORS = 8;

PLOT:

TYPE = PLOT3;

OUTPUT:

TECH11;

***B. Mplus syntax to explore demographic covariates of three-profile solution***

VARIABLE:

NAMES = ZWBEM ZWBOUT ZPHWB AGE BOY OTHERGEN LANG SES

DIFFEM VERBEM HIDE BODY ATT ANA SB MALS PADAY PAHRLO

PAHRMOD PASEDLO PASEDMOD CPROB1 CPROB2 CPROB C;

USEVARIABLES = C AGE BOY OTHERGEN LANG SES;

NOMINAL = C;

MISSING = *;

CLASSES = C1(3);

ANALYSIS:

TYPE = MIXTURE;

STARTS = 0;

ALGORITHM=INTEGRATION;

INTEGRATION=MONTECARLO;

MODEL:

%OVERALL%

C1 ON AGE BOY OTHERGEN LANG SES;

AGE BOY OTHERGEN LANG SES; !FIML

%C1#1%

[C#1@8.547];

[C#2@6.662];

%C1#2%

[C#1@-0.323];

[C#2@2.479];

%C1#3%

[C#1@-11.704];

[C#2@-2.413];

OUTPUT:

cinterval;

***C. Mplus syntax to explore physical activity covariates of three-profile solution***

VARIABLE:

NAMES = ZWBEM ZWBOUT ZPHWB AGE BOY OTHERGEN LANG SES

DIFFEM VERBEM HIDE BODY ATT ANA SB MALS PADAY PAHRLO

PAHRMOD PASEDLO PASEDMOD CPROB1 CPROB2 CPROB C;

USEVARIABLES = C PADAY PAHRLO PAHRMOD PASEDLO PASEDMOD;

NOMINAL = C;

MISSING = *;

CLASSES = C1(3);

ANALYSIS:

TYPE = MIXTURE;

STARTS = 0;

ALGORITHM=INTEGRATION;

INTEGRATION=MONTECARLO;

MODEL:

%OVERALL%

C1 ON PADAY PAHRLO PAHRMOD PASEDLO PASEDMOD;

PADAY PAHRLO PAHRMOD PASEDLO PASEDMOD; !FIML

%C1#1%

[C#1@8.547];

[C#2@6.662];

%C1#2%

[C#1@-0.323];

[C#2@2.479];

%C1#3%

[C#1@-11.704];

[C#2@-2.413];

OUTPUT:

cinterval;

***D. Mplus syntax to explore educational covariates of three-profile solution***

VARIABLE:

NAMES = ZWBEM ZWBOUT ZPHWB AGE BOY OTHERGEN LANG SES

DIFFEM VERBEM HIDE BODY ATT ANA SB MALS PADAY PAHRLO

PAHRMOD PASEDLO PASEDMOD CPROB1 CPROB2 CPROB C;

USEVARIABLES = C DIFFEM VERBEM HIDE BODY ATT ANA SB MALS;

NOMINAL = C;

MISSING = *;

CLASSES = C1(3);

ANALYSIS:

TYPE = MIXTURE;

STARTS = 0;

ALGORITHM=INTEGRATION;

INTEGRATION=MONTECARLO;

MODEL:

%OVERALL%

C1 ON DIFFEM VERBEM HIDE BODY ATT ANA SB MALS;

DIFFEM VERBEM HIDE BODY ATT ANA SB MALS; !FIML

%C1#1%

[C#1@8.547];

[C#2@6.662];

%C1#2%

[C#1@-0.323];

[C#2@2.479];

%C1#3%

[C#1@-11.704];

[C#2@-2.413];

OUTPUT:

cinterval;
